# Where and What Kind—A Better Understanding of Local and Landscape Features in Planning the Urban Flower Meadows for Supporting Bee Communities

**DOI:** 10.1002/ece3.71376

**Published:** 2025-06-17

**Authors:** Agata Kostro‐Ambroziak, Anna Sobieraj‐Betlińska, Piotr Szefer, Urszula Suprunowicz, Bartosz Ulaszewski, Artur Pliszko, Justyna Burzyńska, Beata Charubin, Karolina Wróbel, Karolina Mierzyńska, Daniel Kozikowski, Edyta Jermakowicz

**Affiliations:** ^1^ Department of Zoology and Genetics, Faculty of Biology University of Bialystok Białystok Poland; ^2^ Department of Environmental Biology, Faculty of Biological Sciences Kazimierz Wielki University in Bydgoszcz Bydgoszcz Poland; ^3^ Faculty of Science University of South Bohemia Ceské Budejovice Czech Republic; ^4^ Institute of Entomology Czech Academy of Science Ceské Budejovice Czech Republic; ^5^ Faculty of Biology, The Włodzimierz Chętnicki Biological Science Club University of Bialystok Białystok Poland; ^6^ Doctoral School of University of Bialystok Białystok Poland; ^7^ Department of Genetics, Faculty of Biological Sciences Kazimierz Wielki University in Bydgoszcz Bydgoszcz Poland; ^8^ Department of Taxonomy, Phytogeography and Palaeobotany, Institute of Botany Jagiellonian University Kraków Poland; ^9^ Department of Plant Biology and Ecology, Faculty of Biology University of Bialystok Białystok Poland

**Keywords:** designed landscapes, pollinators, urban ecology, urban ecosystems, urban greenspaces

## Abstract

Cities are growing ecosystems in the modern world. Due to their heterogeneity, urban areas have multifaceted influences on organisms, including bees. However, in many specific city greenspace designs and management implementations, our understanding of their functionality remains limited. This is also true for urban flower meadows (UFMs). We extensively examined UFMs in three large cities to answer the following question: What features of UFMs and their surroundings (urban matrix) are the most important in supporting bees in cities? Our multifaceted approach revealed that the mosaics of habitats surrounding UFMs are at least as necessary to support bees as the local features of UFMs. An abundance of bees responded positively to the number of flowering units and the blue and yellow colors of flowers, and increased cover of industrial areas, green urban areas, and pastures in a 100‐m buffer. Increasing the cover of the continuous urban fabric in all buffer zones (100, 300, and 500 m) positively affected bee species richness and abundance. Due to the lack of design guidelines for urban flower meadows, our results are helpful for the further planning of UFMs to optimize bee‐friendly areas in urban landscapes.

## Graphical abstract

Our multifaceted approach revealed that the mosaics of habitats surrounding urban flower meadows are at least as necessary to support bees as the local features of UFMs. An abundance of bees responded positively to the number of flowering units, the blue and yellow colors of flowers, and increased cover of industrial areas, green urban areas, and pastures in a 100‐m buffer. Our results are helpful for the further planning of UFMs to optimize bee‐friendly areas in urban landscapes.

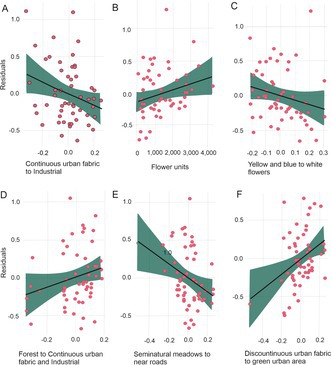

## 1 Introduction

Ecosystems of the urban areas are expanding in the modern world (Seto et al. 2012; United Nations Department of Economic and Social Affairs 2019), which has multifaceted impacts on organisms (Alberti et al. 2017; Jones and Leather 2012). An increasing number of studies have indicated that urban landscapes can support biodiversity (Belaire et al. 2023), positively affecting pollinator taxa, including wild bees (Baldock et al. 2015). There is abundant evidence of a loss of diversity and shrinking ranges in some wild bee species (Biesmeijer et al. 2006; Lebuhn et al. 2013; Ollerton 2017), and urbanization is identified as one of the reasons for this situation (Fortel et al. 2014; Harrison and Winfree 2015; Theodorou et al. 2020; Vimal et al. 2012). However, the impact of urbanization on bee abundance and species richness remains inconclusive. Some studies have shown a positive impact (McFrederick and LeBuhn 2006; Osborne et al. 2007; Theodorou et al. 2016; Wilson and Jamieson 2019) or no impact (Egerer et al. 2020; Felderhoff et al. 2023; Sobieraj‐Betlińska and Twerd 2024). Positive results for bee biodiversity are typically associated with low‐to‐medium levels of urbanization (Wenzel et al. 2020, 2022). In recent years, studies have explored the influence of urbanization on diverse functional traits of bees (Brasil et al. 2024; Cohen et al. 2022; Gruver and CaraDonna 2021; Sobieraj‐Betlińska and Twerd 2024). Urbanization can change bee community composition through novel combinations of available plant species (Ayers and Rehan 2021), increasing the abundance or species richness of cavity‐nesting bees and generalist species (Cane et al. 2006; Matteson et al. 2008) or thermophilic and xerothermic bee species (Banaszak‐Cibicka 2014).

Cities are heterogeneous ecosystems, and there is limited knowledge of how specific design and management choices impact environmental benefits within such a highly modified landscape (Belaire et al. 2023). Baldock et al. (2019) showed that residential and community gardens are urban pollinator ‘hotspots’. In this context, introducing species‐rich herbaceous communities into cities is a valuable ecological model for managing open urban greening. Revegetating urban habitats with flower‐rich meadows, referred to as semi‐natural, multi‐species flower meadows, is relatively recent but has been established in various European countries (Bretzel et al. 2016; Mody et al. 2020). The greatest experience in this area has been in the UK and Germany, where commonly named ‘urban flower meadows’ (UFMs) have been established on a large scale since 2015 (Hoyle et al. 2018). The establishment of this type of flower‐rich meadow in urban areas has covered hundreds of hectares of land. These practices are seldom formalized, and there is a degree of flexibility concerning location and species composition. The selection of plant species that are sown in such areas primarily relies on habitat and aesthetic considerations, as determined by green space managers. Some forms of standardization of wildflower meadows are implemented in England and have included the design of seed mixes, particularly towards farmlands, e.g., “Nectar flower mix” or “Legume rich sward” that have targeted a certain group of pollinators (Department for Environment, Food and Rural Affairs 2014).

The studies emphasized the importance of various meadow features, with a particularly high abundance and richness of plant species (Penberthy et al. 2023; Theodorou et al. 2020), flower color diversity (Hoyle et al. 2018), high diversity of floral resources (generalist vs. specialist plant species) (Howlett et al. 2021; Uyttenbroeck et al. 2017; Vrdoljak et al. 2016), and herbaceous plant height (Albrecht et al. 2023; Dylewski et al. 2024; Felderhoff et al. 2023). The inclusion of particular plant species in sown urban meadows enhances their support for a richer pool of pollinators and more specific pollinators (Griffiths‐Lee et al. 2022). Current data indicate that planting native herbaceous plants can support a rich and abundant bee population (Blackmore and Goulson 2014; Bretzel et al. 2016; Wenzel et al. 2020). In contrast, other studies have emphasized that honeybees may respond positively to exotic flower abundance. Nevertheless, native bees may not have a relationship with plant species of geo‐historical origin (Bendel et al. 2019). The available spatial data indicate that several factors, including meadow types (Norton et al. 2019) and connectivity of green infrastructure (Pla‐Narbona et al. 2022; Smith et al. 2006), have a positive impact on bee communities. The number and quality of urban green spaces on a landscape scale, along with the availability of nesting resources and flowering plants, are of great importance in maintaining bee diversity (Wenzel et al. 2020).

Understanding how UFMs can be optimized to better support bees in cities, particularly in the context of many national strategies to conserve and restore pollinators (Stout and Dicks 2022), is essential. Thus, our study aimed to identify which local features (i.e., flower colors, plant origin, greenery, bare soil) of UFMs and their surroundings (various components of urban matrix) affected bee abundance and species richness. Specifically, we aim to answer the following questions: (1) Do other features of meadows, apart from flower resources, significantly affect bees? (2) Is the size of UFM important in supporting bees? (3) Which/how various forms of urban greenery in the surroundings of UFMs promote bees? and (4) Is urban infrastructure a limiting factor for bees on UFMs? Such a deeper understanding of how bees respond to diverse urban flower meadows in different urban landscapes can lead to the development of valuable cues for further planning of UFMs.

## 2 Materials and Methods

### 2.1 Study Area

**FIGURE 1:**
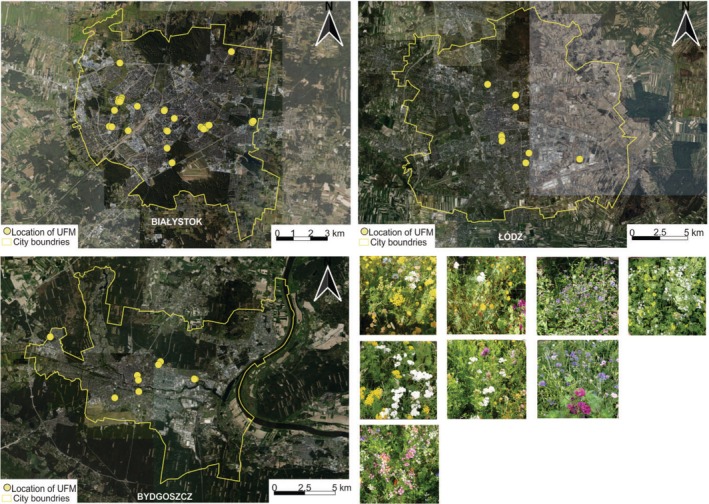
Maps showing locations and examples of the structure of the studied urban flower meadows.

Field research was conducted in 2021 in three major cities (> 290,000 inhabitants): Białystok (BI) (53°07′59″ N 23°09′51″ E), Bydgoszcz (CB) (53°7′24.6″ N 18°0′27.43″ E), and Łódź (EL) (51°45′00″ N 19°28′00″ E), located in different parts of northern, central, and east Poland (Figure 1). The cities differ in the share of overall green areas (from 35.1% in Białystok to 51.4% and 55.7% in Bydgoszcz and Łódź, respectively (Łachowski and Łęczek 2021) and in the total area of urban flower meadows (90,000, 12,000, and 5,000 m^2^ in Białystok, Bydgoszcz, and Łódź, respectively). The study was conducted on all open green areas designed as urban flower meadows (*N* = 55) that were sown 1–3 years before our study. They differed in occupied area and cover from about 80 to 5,000 m^2^. Both multi‐species meadows and monocultures, with a clear dominance of 1–2 species, were included. They were located within parks, street lines, abandoned areas, and city centers (Figure 1).

### 2.2 Bee Sampling

Bees were caught in randomly located 55 plots with standardized size (2 × 2 m), one plot per UFM, once, from June 23 to July 3, 2021, during the peak of flower blooming. We selected this point because it represents the period when most flowering plants bloom in all studied flower meadows, both in multi‐species meadows and monocultures; in the latter case, re‐collection of bees in the later parts of the season could not be practiced due to their short flowering period. Furthermore, as bee species richness and abundance in urban areas remain relatively stable throughout the year, sufficient data can be gathered, even with brief sampling periods (Buchholz et al. 2020; Leong et al. 2016; Rakosy et al. 2022). Due to the great variability in the size of the flower meadows, all bees from the plots were collected for 20 min using an entomological net to standardize the samples (Zajdel et al. 2025). To avoid edge effects, sampled plots were at least 10 m from the meadow border (Andrieu et al. 2018). Bee sampling was conducted when the weather was favorable for bee activity, that is, at a temperature exceeding 16°C (average of 25°C across site sampling events), with no or little wind (< 3 on the Beaufort scale), and when the cloudless sky was at least approximately 70% (Krauss et al. 2009). Bumblebees and other bee species that were easily distinguished were identified as alive in the field. Other bee specimens were collected for species identification and deposited in the Laboratory of Insects Evolutionary Biology and Ecology, University of Bialystok. Identification was accomplished using various keys (Data S1). The nomenclature followed Kuhlmann et al. (2012).

Functional traits for the collected species were compiled from published literature (Banaszak‐Cibicka and Dylewski 2021; Gathof et al. 2022; Sobieraj‐Betlińska et al. 2023; Twerd et al. 2021). For each species, social behavior (solitary, eusocial, or cleptoparasitic), nest substrate (cavity, soil, or hive), floral specificity (oligolectic or polylectic), and body size (small < 8 mm, medium 8–15 mm, or large > 15 mm) were determined (Table S1).

The Regional Directorate for Environmental Protection in Białystok, Bydgoszcz, and Łódź issued the relevant permissions for studies (decision nos WPN.6401.220.2021.MK, WOP.6401.5.15.2021.MO, and WPN.6401.210.2021.BWo).

### 2.3 UFM Variables

The structure of the particular UFM planted in the studied cities was almost homogeneous. Thus, we selected a representative part of the given UFM patch, where a plot of 4 m^2^ was established, with a grid of 16 squares (0.5 × 0.5 m). UFMs were characterized based on their structure. Described variables comprised the pertained flower resources (C1–C4), flower color display (C5–C13), plant origin (C14–C17), and vegetation structure (C18–C21). The area of the given UFM (C22) was also included in the data. Detailed floristic studies were conducted for all the UFMs. We identified all vascular plant species (C1) and compiled lists of entomogamous plant species that were in bloom at the time of the field investigation (C2); anemogamous/autogamous plant species were classified within the C3 variable. Plant identification was based on morphological features described by Rutkowski (2022) and Snowarski (2024). The plant nomenclature was given according to the International Plant Names Index (IPNI 2024). The pollination mode followed the Ecological Flora of Britain and Ireland (Fitter and Peat 1994) and Pladias (2014). We used quantitative estimations to collect data on potential bee forage resources. We assessed the number of ‘flower units’ (C4) on the study plots. One ‘flower unit’ was counted as a single flower or single dense inflorescence with small flowers (< 1 cm), in the case of the umbel (e.g., *Daucus carota* ), head (e.g., *Trifolium repens* ), or capitulum (e.g., *Cota tinctoria* ), and was defined according to Dicks et al. (2002) as the visual display from which a medium‐sized bee will fly to the next unit rather than walk. We assigned each flower unit to one of the seven color classes (white, yellow, white‐yellow, pink, blue, purple, and red) and calculated the frequency of particular colors in plots (variable C5–11), the sum of the colors of blooming flowers (C12), and color heterogeneity (C13) according to the Shannon index *SHDI* = 1 − Σ*p*_*i*_ × *p*_*i*_ (Shannon and Weaver 1998), where *p*_*i*_ is the contribution of *i*‐color to the sum of colors in the given plot. *SHDI* = 0 indicates a one‐colored flower meadow. We evaluated the naturalness level of each UFM based on the frequency of plant species belonging to each geographical‐historical group: apophytes (C14), native species occurring in semi‐natural and anthropogenic habitats; archaeophytes (C15), alien species established before 1492; neophytes (C16), alien species established after 1492; and diaphytes (C17), casual alien species not established in the Polish flora (Tokarska‐Guzik et al. 2012). UFMs with the highest proportions of apophytes and archaeophytes were described as the most natural, in contrast to meadows with a high proportion of neophytes and diaphytes. We also estimated the contributions of grasses (C18), dicotyledonous plants (C19), and bare soil (C20) to each UFM. These measures were used to calculate habitat heterogeneity (C21) according to the Shannon index *SHDI* = 1 − Σ*p*_*i*_ × *p*_*i*_, where *p*_*i*_ is the contribution of a particular type of ground cover.

The area size (in m^2^) of each UFM (C22) was determined based on aerial photographs on a scale of 1:2500, using the open‐source Geographic Information System QGIS Desktop 3.1 (QGIS Development Team 2020) and direct field research.

### 2.4 Landscape Variables—Urban Matrix Features

All GIS‐related tasks were performed using QGIS software version 3.10 (QGIS Development Team 2020). Measurements of the area for all UFMs obtained during the biodiversity survey were converted into polygons in the *.*shp* format. The correctness of the polygons was verified using the Orthophotomap of Poland (https://www.geoportal.gov.pl/dane/ortofotomapa). For each meadow, three buffer zones were established with a width of 100, 300, and 500 m measured from the border of each area (Badiane et al. 2024; Martins et al. 2017; Rivers‐Moore et al. 2023) (Figure S1A–C). Close proximity to nests and foraging resources within a radius of a few hundred meters (from 100 to 300 m) is crucial for maintaining solitary bee populations (Greenleaf et al. 2007; Zurbuchen et al. 2010; Wright et al. 2015). The maximum foraging distances of bees at the species level may be longer, i.e., 1100–1400 m, but it appears that such long distances apply only to single females (Zurbuchen et al. 2010). Besides, large species of social bees, such as bumblebees, are able to forage within a radius of more than 1000 m from their nest (Steffan‐Dewenter and Kuhn 2003), but usually fly much shorter distances (Geib et al. 2015). In each buffer zone, 10,000 randomly distributed points were created with a minimal distance of 1 m from each other. Urban Atlas 2018 maps (Copernicus 2020) describing 20 various types of urban areas (Table S2) were downloaded from Copernicus servers for Białystok, Bydgoszcz, and Łódź. For each set of points, two types of information were collected using the Point Sampling Tool plug (version 0.5.3; https://github.com/borysiasty/pointsamplingtool): (1) information on overlapping buffer zones of other flower meadows; (2) information on types of urban areas within the buffer. The results were saved to *.*csv* files and summarized with a custom bash script for further statistical processing.

### 2.5 Statistical Analysis

To assess the representativeness of the collected bees in urban flower meadows and to analyze their species richness, we generated rarefaction curves (Gotelli and Colwell 2001) using the *iNEXT* package (Hsieh et al. 2016). To estimate true species diversity, we used the Chao estimators of three diversity estimates: number of species (species richness), Shannon's, and Simpson's index (Chao 1984).

We used generalized linear models to test the impact of local (UFMs) and urban matrix (coverage of various landscape classes in the surroundings of UFMs) feature variables on bee abundance and species richness of UFMs. Local and landscape factors of the studied meadows were chosen a priori due to their known effects on bee abundance and species richness (e.g., Buchholz et al. 2020; Twerd et al. 2022). Initially, we performed Moran's *I* test to evaluate for spatial correlation between the sites. We did not find any spatial correlation between UFMs (Moran's test for species richness: *I* = 0.068; *p* = 0.412, and for abundance, *I* = 0.053; *p* = 0.485). Therefore, individual UFMs were treated as spatially independent observations. To reduce the dimensionality of our explanatory variables, we performed PCA on the surrounding characteristics and used the first six PC axes for further statistical analysis (Figure S2). The six selected PC axes accounted for approximately 90% of the variability between the UFMs. For the same reason, we independently performed PCA on seven color characteristics of each meadow and also selected the first two PC axes, which accounted for approximately 73% of the variation in colors between the sites in the final model (Figure S3).

We performed a backward selection of variables (meadow characteristics C2–C4, C13, C15–C17, C20 and PC axes for flower color) while controlling for the surroundings (i.e., keeping logarithm of the UFMs area and all six PC axes at each model selection step) to evaluate which local meadow characteristics are responsible for the bee abundance and species richness. We used the negative binomial and Poisson distributions in the GLM to model the residuals of the abundance and species richness, respectively. The variance inflation factors (VIFs) were calculated for the final model to evaluate potential colinearity. Calculations were made using vegan (Oksanen et al. 2022) and the *MASS* library (Venables et al. 2002).

The urban matrix features differed in characteristics between the cities (Figure S4). This allowed us to analyze UFMs in different surroundings, which reflects the wide range of landscape variables in which UFMs are planted. This decision not to include city ID as a factor in the statistical models was further supported by the overlapping histograms for environmental variables and the fact that species richness and abundance of bees were the same in all three cities (Figure S5).

The surrounding characteristics of the studied UFMs varied. Because of the unbalanced or low cluster sizes (*K*‐clustering procedure) we decided to only control for the surroundings in our model by performing PCA analysis and including the first six axes during the backward selection model: PC1—from forests (in all buffer zones) to industrial areas and continuous urban fabric (surface land [s.l.]: > 80%), PC2—from roads in a 100‐m buffer zone to discontinuous urban fabric in all buffer zones, PC3—continuous urban fabric (s.l.: > 80%) in a 100‐m buffer zone to industrial areas concentrating in a 100‐m buffer zone (gradient goes through 500‐m, 300‐m, towards 100‐m buffer zones), PC4—from pastures in all buffer zones to forests in all buffer zones, PC5—from pastures to roads in 100‐m buffer zones, PC6—from discontinuous urban fabric in all buffer zones to concentrating green urban areas (gradient goes through 500‐, 300‐, towards 100‐m buffer zones).

Statistical analyses were performed with the R programming language version 4.2.2 using only functions provided in the cited libraries (R Core Team 2022).

## 3 Results

### 3.1 Flower Meadow Structure

The examined flower meadow patches displayed a broad range of values (Figure S5), reflecting local characteristics and distinctiveness. A total of 230 flowering plant species were recorded, of which 76.5% were entomophilous. The floral composition, number of plant species (11–53), and proportion of entomophilous plants (6–38) varied significantly throughout the study area. The sum of the flower units ranged widely, from almost exclusively grassy meadows with nearly no flowers to the highest floral densities, reaching 4277 floral units per study plot. The studied UFMs differed in naturality; most (65.5%) primarily comprised (> 80%) plant species perceived as native to this geographical region, and diaphytes were absent in 38.2% of the studied UFMs. The UFMs differed in color composition from one‐colored (*SHDI* = 1) to heterogeneous meadows with a variable share of all seven color classes (*SHDI* = 1.24–1.52).

The study meadows differed in structure, conditioned by the proportions of grasses, forbs, and bare soil. When plant cover was dominated by forbs (> 70%), grasses did not exceed 10%–20%. In most cases, the bare soil did not exceed 10%–15% of the study plots; only in eight cases was > 15% of the plot surface occupied by exposed soil fragments (Figure S5).

### 3.2 Species Richness and Functional Diversity Components of Bees

We report 50 species of bees (Hymenoptera: Apoidea: Apiformes) from 17 genera, represented by 654 individuals. The recorded bees represented the following families in terms of species richness and abundance: Apidae (15 species, 404 individuals), Halictidae (7, 16), Megachilidae (16, 97), Andrenidae (5, 21), Colletidae (5, 93), and Melittidae (2, 23) (Table S3). The species richness and abundance of bees in the UFMs were similar in all three cities (Figure S6). The species accumulation curves did not reach saturation, indicating that some species remained undetectable. The Chao estimators suggested 50 species for Białystok (estimated completeness of 70%), 41 for Bydgoszcz (estimated completeness of 51%), and 37 for Łódź (estimated completeness of 68%) (Table S4). The bee species recorded in the UFMs represented various functional groups (Table 1 and Table S3).

**TABLE 1:** Characteristics of the total catch of bees found in urban flower meadows in three Polish cities.

Functional traits of bees		Bee species		
		*S*	% *S*	Example
Social behavior				
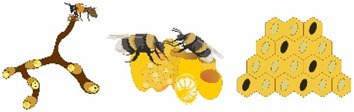	Solitary	32	64	*Colletes similis*
	Eusocial	17	34	*Bombus lapidarius*
	Cleptoparasitic	1	2	*Bombus bohemicus*
Nest substrate				
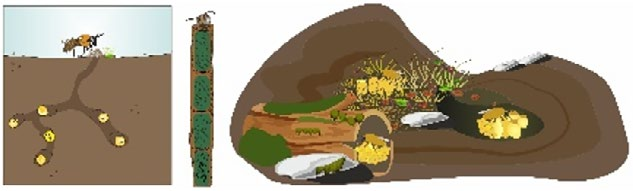	Soil	23	46	*Melitta leporina*
	Cavity	13	26	*Anthidium manicatum*
	Hive	13	26	*Bombus pascuorum*
Floral specificity				
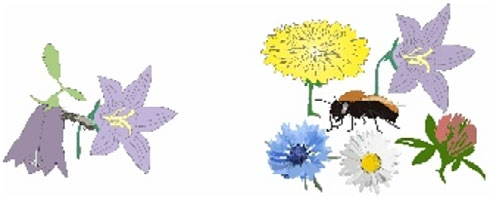	Oligolectic	12	24	*Anthophora furcata*
	Polylectic	37	74	*Andrena flavipes*
Body length (mm)				
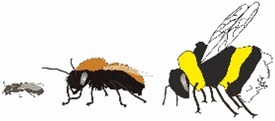	Small	11	22	*Heriades truncorum*
	Medium	33	66	*Megachile willughbiella*
	Large	6	12	*Bombus hortorum*

Abbreviation: *S*, number of species.

### 3.3 Influence of Local and Urban Matrix Features on Bee Abundance and Species Richness of UTMs

**FIGURE 2:**
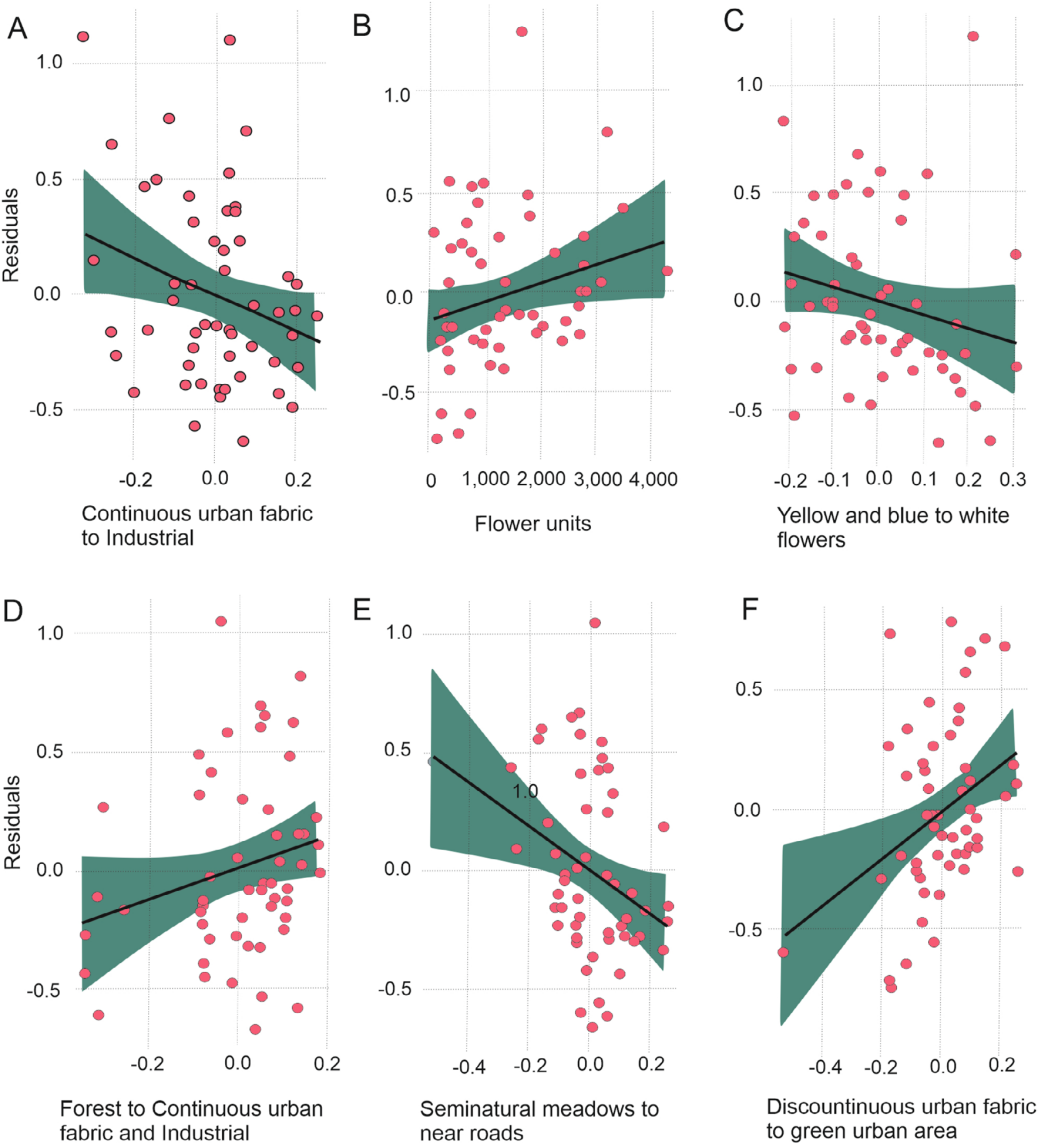
Partial correlations for the significant local floral meadow variables for bee species richness (A) and abundance (B–F). Residuals were taken from a model without an explanatory variable. For example, residuals for species richness were taken from the model presented in Table 2 but without the PC3 variable. Correlations better represent the effect of a variable on abundance and species richness when other factors (covariates) are being controlled. A—species richness explained by PC3, B—Abundance explained by a number of flower units, C—Abundance explained by PC2 for the flower color, and D–F—Abundance explained by the PC1, PC5, and PC6, respectively.

The area of UFMs, the number of flower units, the contribution of diaphytes and bare soil, and the second PC axis for flower unit color (representing a gradient of blue and yellow to white flowers) were all components of the final model for bee abundance on UFMs (Table 2, Figure 2). The number of flower units positively affected the bee abundance. An increase of 100 flower units in the whole meadow would result in an increase of approximately 7% in bee abundance. The second PC axis for plant color had a negative effect. An increase of one standard deviation towards blue and yellow flower coverage resulted in an approximately 26% increase in bee abundance. PC1, PC5, and PC6 of the surrounding area also significantly affected bee abundance: PC1 and PC6 positively, and PC5 negatively. Therefore, surroundings with a higher cover of industrial areas and continuous urban fabric in all buffer zones (PC1, approximately 23% increase per 1 SD), increased coverage of pastures in 100‐m buffer zones (PC5, approximately 20% per 1 SD), and green urban areas in all buffer zones (PC6, approximately 22% per 1 SD) appear to benefit wild bee abundance.

**TABLE 2:** Results of the backward selection for the negative binomial model for bee abundance (number of observations = 55, *R* ^2^ Nagelkerke = 0.825).

Parameter	Estimate	SE	*Z*	*p*
(Intercept)	2.727e+00	2.898e‐01	9.412	< 2e‐16
Area of UFMs	−8.193e‐02	4.415e‐02	−1.856	0.063470
Diaphytes	−5.723e‐03	3.850e‐03	−1.487	0.137102
Number of flower units	1.286e‐04	4.614e‐05	2.787	**0.005315**
Bare soil	8.507e‐03	5.209e‐03	1.633	0.102439
PC2_color	−1.398e+00	4.185e‐01	−3.341	**0.000836**
PC1	1.262e+00	4.256e‐01	2.964	**0.003034**
PC2	−4.378e‐01	3.794e‐01	−1.154	0.248475
PC3	−4.938e‐01	3.496e‐01	−1.412	0.157852
PC4	−2.417e‐02	3.311e‐01	−0.073	0.941798
PC5	−1.125e+00	3.603e‐01	−3.123	**0.001792**
PC6	1.212e+00	3.632e‐01	3.338	**0.000845**

*Note:* PC1–PC6 were kept in the model to control the variability in bee abundance related to the floral meadows' surroundings. Variables statistically significant at *p* < 0.05 are bolded.

Abbreviation: SE, standard error.

The area of UFMs, color heterogeneity of flower units, and the second PC axis for flower unit color (representing a gradient of blue and yellow to white flowers) were all components of the final model for bee species richness in UFMs (Table 3 and Figure 2). Continuous urban fabric to industrial areas concentrated in 100‐m buffer zones (PC3, approximately 7.6%) appeared to increase bee species richness.

**TABLE 3:** Results of the backward Poisson model for bee species richness (number of observations = 55, *R* ^2^ Nagelkerke = 0.377).

Parameter	Estimate	SE	*Z*	*p*
(Intercept)	1.56595	0.44755	3.499	0.000467
Area of UFMs	−0.04366	0.06204	−0.704	0.481555
Color heterogeneity	0.29229	0.18460	1.583	0.113334
PC2_color	−0.88047	0.56573	−1.556	0.119628
PC1	0.86147	0.52928	1.628	0.103605
PC2	−0.26526	0.51316	−0.517	0.605221
PC3	−1.00529	0.49606	−2.027	**0.042709**
PC4	−0.61518	0.46629	−1.319	0.187062
PC5	−0.66473	0.48966	−1.358	0.174614
PC6	0.14623	0.47192	0.310	0.756665

*Note:* PC1–PC6 were kept in the model to control the variability in bee richness related to the floral meadows' surroundings. Variables statistically significant at *p* < 0.05 are bolded.

Abbreviation: SE, standard error.

## 4 Discussion

Our research considers multiple characteristics of UFMs in terms of their local structures and surroundings (urban matrix). The final models showed that several variables significantly influenced the species richness and abundance of bees recorded in the UFMs. Among these features are those that pertain to the UFM structure, the number of flower units, and the contribution of blue and yellow flowers, which are positively correlated with bee abundance.

The diversity of flowering plant species is the most widely discussed issue regarding resources that are essential for bees as the main group of pollinators (Ballare et al. 2019; Bretzel et al. 2016). Experimental studies analyzing the composition of flower meadows specified this issue and identified two factors: (1) the richness of flowering plant species, which unequivocally enhances the attractiveness of pollinating insects (Blackmore and Goulson 2014; Griffiths‐Lee et al. 2022; Penberthy et al. 2023), and (2) the functional diversity of flower traits (Ayers and Rehan 2021; Banaszak‐Cibicka and Dylewski 2021; Graf et al. 2022; Norton et al. 2019; Uyttenbroeck et al. 2017; Vrdoljak et al. 2016). Gerner and Sargent (2022) showed a 1.5%–2% increase in bee diversity and abundance with the addition of every plant species in urban areas. Thus, in general, a broad spectrum of generalized plant species (with resources available for different groups of pollinators), with a mix of plants and specialized flowers, is the best solution for supporting a high diversity of bee species. Increased flower abundance is thought to increase the pollinator visitation rate (Penberthy et al. 2023; Watson et al. 2022), and our results showed that an increase in flower units positively impacted bee abundance in UFMs. Investigations aimed at detecting plant species that host the greatest bee species richness and abundance, the so‐called central plants in the pollinator–plant network, have led to the identification of several particularly important angiosperm families, which have a high probability of improving bee pollination services (Howlett et al. 2021). A comprehensive analysis conducted by Kuppler et al. (2023) identified 34 herbaceous key plant species and the Asteraceae, Brassicaceae, Lamiaceae, and Fabaceae families as those with the highest bee visitation rates. In these families, we found species with morphologically generalized flowers, and those preferred by only specific groups of bee species. A weighty conclusion that emerged from this study indicates that a key plant species, the most preferred by bees in a given area, generally depends on the composition of the sampled plant community. We did not analyze plant species that specifically attracted bees (the exceptional floristic diversity of individual UFM would not allow for a clear interpretation); however, field observations indicate a particular role of the Asteraceae family with *Achillea millefolium* , *Centaurea cyanus* , or *Cichorium intybus* , as well as Fabaceae with *Lotus corniculatus* and *Melilotus albus* as the most attractive for bees. These observations are consistent with our subsequent results, indicating an increase in bee abundance in UFM patches characterized by a high contribution of blue (e.g., *C. cyanus* and *C. intybus* ) and yellow (e.g., *L. corniculatus* ) flowering plants. Among the different sensory cues used, visual cues are of fundamental importance. Thus, flower color, shape, and pattern are visual signals that allow bees to recognize and discriminate profitable plant species (Giurfa and Lehrer 2001; Nicolson and Thornburg 2007). Referring to our results regarding color preference, the experimental studies (Giurfa and Lehrer 2001) have demonstrated that bees locate blue and yellow flowers twice as fast as red and white flowers, which is linked to their trichromatic vision (UV‐blue‐green). However, bee perception and preferences regarding flower color are modified by plant community composition, associated competition for flower‐visiting insects, and the cognitive abilities of bees (Chittka et al. 2001). As bees use flower patches as sensory cues to find food resources (Dauber et al. 2010), the multi‐flower UFMs act as a long‐distance advertisement of food resources. More effectively, they are resources preferred by the local bees, and the richer and more numerous this bee community is.

Continuity of food resources over time (‘food tape’) is crucial for bees (Ammann et al. 2024; Ogilvie and Forrest 2017; Sobieraj‐Betlińska et al. 2023). Increased resource availability events, which are sporadic and short in duration, but large in scale (i.e., resource pulses), drive population trajectories (Yang et al. 2008). However, when resource gaps exist, resource discontinuities can affect the population over time (Nicholson et al. 2021). Thus, the presence of suitable food plants as nectar and pollen sources during the entire growing season affects bee development, reproduction, and survival (Vaudo et al. 2015). Therefore, promoting plant species with different flowering periods is beneficial, as it extends the attractiveness period to humans and the resources available to pollinators (Hoyle et al. 2018). Therefore, in an urban matrix, it may be more beneficial to establish diverse year‐round meadows than mass‐flowering rapeseed or monocultures, which bloom only for a short time during the vegetation season.

The plots we studied were urban flower meadows managed in this way for 1–3 years. Although it should be mentioned that some studies in urban landscapes have shown a positive effect of the age of green areas on arthropod species richness (McIntyre 2000; Sattler et al. 2010). This was a result of the formation of more ecological niches due to progressive succession, as well as the increasing probability of successful stochastic local immigration. For example, the study by Mody et al. (2020) showed differences in arthropod numbers between old and young wildflower urban meadows. In the first year, the newly created meadows were sparsely vegetated, and arthropods had little time to colonize the new meadow plots (Noordijk et al. 2008). The well‐documented management history of the analyzed urban flower meadow patches, along with the knowledge of the initial state, provides an excellent starting point for further research on the dynamics of bee communities in those places.

Urbanization modifies plant community composition through novel combinations of native species (Harrison and Winfree 2015; Pardee and Philpott 2014) or through the introduction of plants of foreign origin, primarily as ornamental taxa, but also through accidental introduction (Adedoja and Mallinger 2024). Regarding resources particularly attractive to wild bees, there is an increasing focus on the geographical‐historical matching of flower resources to native bee species (Pardee and Philpott 2014), which has particular implications when discussing the design of plant species compositions for UFMs. The analyzed UFMs displayed significant variation in the ratio of alien to native plant species. Consequently, for some of them, we can assert that they are natural meadows entirely devoid of exotic species, whereas there were UFMs where exotic species constituted > 30%–40% of all plants. Regardless of this result, there is a need to promote the naturalness of UFMs, in line with other studies, including those related to backyard gardens and other urbanized areas where native flowers are considered to have a noticeably positive impact on bee communities (Ballare et al. 2019; Fenoglio et al. 2023; Griffiths‐Lee et al. 2022; Pardee and Philpott 2014). Other authors have highlighted the necessity of planting native plant species, although with some selection of exotic flowers, which can provide additional food resources for generalist and specialized bees, which could contribute to extending the flowering season (Hoyle et al. 2018; Salisbury et al. 2015). A similar effect can also be achieved through the preservation of semi‐natural ruderal areas, along with a network of backyard gardens enhanced by diverse UFMs, which may become an important local strategy for promoting the conservation of bees. The next step could be the approval of spontaneous vegetation for ornamental purposes, which is now becoming widespread and more accepted as a fundamental landscape design tool (Farruggia et al. 2022; Nichols et al. 2019). The presence of preferred plant genera, families, or trait groups is more important for the persistence of specialist pollinators than plant geographic origins (Harrison and Winfree 2015; Wenzel et al. 2020). However, there is still an open question as to how non‐native species, together with specifically managed vegetation in urban habitats, influence community‐level plant phenology and, consequently, pollinator phenology in the long term (Adedoja and Mallinger 2024; Harrison and Winfree 2015).

In urban bee fauna, generalist feeders, such as honeybees, bumblebees, or sweat bees (*Lasioglossum*), can be dominant groups (Ayers and Rehan 2021; Rahimi et al. 2022), and specialist (oligolectic) pollinators are sometimes absent or depleted (Cane et al. 2006; Matteson et al. 2008). Furthermore, in urban ecosystems, pollen specialists may be more vulnerable to urbanization or local extinction (Buchholz and Egerer 2020). The bee communities in the studied urban meadows constituted mostly polylectic species; however, the proportion of oligolectic species was relatively high (25%). In contrast, bee studies at locations within an urban matrix representing the wide range of urban environments of Poznań reported only 15% of oligolectic species (Banaszak‐Cibicka and Żmihorski 2012).

In addition to foraging resources (quantity and quality), nesting places are crucial for supporting bees (Blackmore and Goulson 2014; Bretzel et al. 2016). Female bees undertake frequent flights between their nests and host plants to collect pollen and nectar for their larvae. They generally forage for food at short distances from their nest (Gathmann and Tscharntke 2002). Therefore, the management of urban areas focusing on bees requires that nesting sites and foraging resources are abundant and as close together as possible. Interestingly, 46% of the bee species recorded on the UFMs constituted species that nest in soil, which are typically underrepresented in urban environments, considering that bare soil for endogeic bees becomes more limited in highly urbanized areas, largely covered with impervious surfaces (Gathof et al. 2022). This contrasts with many studies from other regions where more species of cavity‐nesting bees have been reported in the UK (Bates et al. 2011), New York (Matteson et al. 2008), and Brazil (Zanette et al. 2005). Our results correspond to studies showing that residential gardens, urban parks, and grassland habitats may promote ground‐nesting bee species, as these green spaces often contain bare soil and stem nesting substrate (Ayers and Rehan 2021; Neumann et al. 2024).

The surrounding UFMs are essential factors that influence bees. Interestingly, the surroundings with a higher cover of industrial areas and continuous urban fabric (> 80% of the land surface) in all buffer zones (100, 300, and 500 m) benefit bee abundance, and the latter feature increases bee species richness in UFMs. This is consistent with studies that have shown the positive effects of urbanization on bees (McFrederick and LeBuhn 2006; Osborne et al. 2007; Theodorou et al. 2016). Poole et al. (2024) found that public urban green spaces enhanced with wildflower meadows and pollinator‐friendly ornamental plants mitigated the negative impacts of urbanization on pollinators, particularly *Bombus* spp. However, most studies have indicated a decline in bee species richness and/or abundance with increasing urbanization (Bates et al. 2011; Bennett and Lovell 2019; Glaum et al. 2017; Hernandez et al. 2009; Zanette et al. 2005), which reduces or eliminates the availability of suitable habitats for bees, limits their food and nesting sites, or restrict their movement. Increasing the impervious surfaces decreases soil‐nesting bees (Birdshire et al. 2020; Geslin et al. 2013) but minimally impacts aboveground nesting bees (Pfeiffer et al. 2023) because of cracks and holes; this area can provide essential aboveground nesting resources for bees. The specific impact of impervious surface cover on bees is complex, as it shows differences in relation to many unique geographical, historical, and economic factors (Fauviau et al. 2022; McKinney 2008).

Increasing green urban areas in cities can support the species richness and abundance of bees (Ayers and Rehan 2021; Tonietto et al. 2011), which corresponds with our results on bee abundance in all buffer zones of the UFMs. The urban green areas in the examined buffers included gardens, parks, squares, and cemeteries. As various types of green urban areas can provide essential foraging and nesting resources for bees (Dietzel et al. 2024; Jacobs et al. 2023; Normandin et al. 2017; Sobieraj‐Betlińska and Twerd 2024), they are also important features of the surroundings at different distances from the UFMs. The size of the green area required to support bees remains poorly understood (Lepczyk et al. 2017). Similar to the UFMs we studied, several other studies have not reported a statistically significant effect of urban green space size on the species richness, abundance, and community composition of bees (Gunnarsson and Federsel 2014; Sobieraj‐Betlińska and Twerd 2024; Twerd et al. 2022). For example, Griffiths‐Lee et al. (2022) showed that sown ‘mini meadows’ (4 m^2^) increase the diversity of pollinating insects in domestic gardens and allotments. Other British studies (Griffiths‐Lee et al. 2022) emphasized that in an urban matrix, even a small area of wildflower meadow had significantly higher pollinator abundance and species richness than comparable amenity grasslands. Thus, it is necessary to use even the smallest areas in the city to establish flower‐rich meadows to support local bee communities.

The contribution of the landscape variable defined according to the Urban Atlas (Copernicus 2020) as pasture around the studied flower meadows positively influenced overall bee abundance. In our study, this variable mainly included semi‐natural grasslands at various succession stages, including wetlands, which are rare and threatened in European landscapes (Gigante et al. 2024; Moroń et al. 2008; Shipley et al. 2024). In cities, it was anticipated that higher species abundance and richness would occur in habitats closer to natural areas (Jones and Leather 2012). The studies have found that preserving grassland habitats, meadows, or wastelands in cities promotes bee diversity and mitigates the negative impact of human activities on the urban landscape (Dylewski et al. 2024; Sobieraj‐Betlińska and Twerd 2024; Venn et al. 2023). Notably, bees in urban areas respond to different land uses at different scales (Fischer et al. 2016; Weber et al. 2023). In the current study, total bee abundance benefited from the proportion of pastures within the smallest radius, i.e., 100 m. Nearby grassland habitats may be the most beneficial to solitary bees, which are forced to forage for short distances (Zurbuchen et al. 2010).

## 5 Conclusion and Recommendations for Planning and Management of UFMs

UFMs can support efforts to develop pollinator‐friendly green spaces within urban green infrastructure; however, we should consider the local and surrounding features of the planned UFMs. It appears that the heterogeneity of urban habitats surrounding UFMs is at least as important to bees as the local features of UFMs. When selecting a seeding material that is friendly to bees, managers of green areas in cities should aim to establish multi‐flower meadows, rich in flower units, but also in blue and yellow flowers, that are in bloom all season long. Furthermore, we recommend planting UFMs (independent of their size) near other green urban areas (such as parks, gardens, and semi‐natural meadows), industrial areas, and continuous urban fabric. This will improve the connectivity of these habitats in the urban matrix and support the local bee community by supplying foraging and nesting resources within a short distance.

## Author Contributions

**Agata Kostro‐Ambroziak:** conceptualization (lead), formal analysis (lead), funding acquisition (lead), investigation (equal), project administration (lead), supervision (lead), writing – original draft (lead), writing – review and editing (lead). **Anna Sobieraj‐Betlińska:** data curation (lead), investigation (equal), methodology (lead), resources (lead), writing – original draft (equal), writing – review and editing (equal). **Piotr Szefer:** formal analysis (lead), visualization (equal), writing – original draft (equal). **Urszula Suprunowicz:** investigation (equal), project administration (supporting), resources (equal). **Bartosz Ulaszewski:** formal analysis (equal), methodology (equal), visualization (lead), writing – original draft (supporting). **Artur Pliszko:** data curation (equal), resources (lead), writing – original draft (supporting). **Justyna Burzyńska:** investigation (equal), project administration (supporting), resources (equal). **Beata Charubin:** investigation (equal), resources (equal). **Karolina Wróbel:** investigation (equal), resources (equal). **Karolina Mierzyńska:** investigation (equal), resources (equal). **Daniel Kozikowski:** investigation (equal), resources (supporting). **Edyta Jermakowicz:** conceptualization (lead), formal analysis (equal), methodology (lead), resources (equal), supervision (equal), visualization (equal), writing – original draft (equal), writing – review and editing (equal).

## Conflicts of Interest

The authors declare no conflicts of interest.

## Supporting information


Data S1.

Figures S1–S6.

Tables S1–S4.

## Data Availability

Ambroziak et. al. Where and What Kind—A Better Understanding of Local and Landscape Features in Planning the Urban Flower Meadows for Supporting Bee Communities. Data availability through Dryad Repository: https://doi.org/10.5061/dryad.z08kprrrc (Apr 08, 2025).
