# Supplementary material for: Where and What Kind—A Better Understanding of Local and Landscape Features in Planning the Urban Flower Meadows for Supporting Bee Communities
Source: Ecol Evol. 2025 Jun 17;15(6):e71376. doi: 10.1002/ece3.71376 (PMC12171940; doi:10.1002/ece3.71376)
Supplement: Supplementary file 1 — Data S1. [file ECE3-15-e71376-s003.docx]

# Supporting information

**Where and what kind – a better understanding of local and landscape features in planning the urban flower meadows for supporting bee communities**

**Information S1**

**Literature used to identify bee species**

Banaszak, J., 1993. Trzmiele Polski. Wyższa Szkoła Pedagogiczna w Bydgoszczy, Bydgoszcz. (in Polish)

Banaszak, J., Romasenko, L., 2001. Megachilid bees of Europe. 2nd edition. Bydgoszcz University of Kazimierz Wielki, Bydgoszcz.

Pawlikowski, T., Celary, W., 2003. Klucze do oznaczania owadów Polski. Część 24. Błonkówki – Hymenoptera. Zeszyt 68a. Pszczołowate – Apidae. Wstęp i podrodzina lepiarkowate – Colletinae. Polskie Towarzystwo Entomologiczne, Toruń. (in Polish)

Pesenko, Y.A., Banaszak, J., Cierzniak, T., 2002. Klucze do oznaczania owadów Polski. Część 24. Błonkówki – Hymenoptera. Zeszyt 68b. Pszczołowate – Apidae. Podrodzina smuklikowate – Halictinae. Polskie Towarzystwo Entomologiczne, Toruń. (in Polish)

Pesenko, Yu.A., Banaszak, J., Radchenko, V.G., Cierzniak, T., 2000. Bees of the family Halictidae (excluding *Sphecodes*) of Poland: taxonomy, ecology, bionomics. Wydawnictwo Uczelniane Wyższej Szkoły Pedagogicznej w Bydgoszczy, Bydgoszcz.

Scheuchl, E., 1995. Illustrierte Bestimmungstabellen der Wildbienen Deutschlands und Österreichs. Band I: Schlüssel der Gattungen und der Arten der Familie Anthophoridae. Eigenverlag, Velden. (in German)

Scheuchl, E., 1996. Illustrierte Bestimmungstabellen der Wildbienen Deutschlands und Österreichs. Band II: Schlüssel der Gattungen und der Arten der Familien Megachilidae und Melittidae. Eigenverlag, Velden. (in German)

Schmid-Egger, C., Scheuchl, E., 1997. Illustrierte bestimmungstabellen der wildbienen Deutschlands und Österreichs unter berücksichtigung der arten der Schweiz. Band 3. Schlüssel der arten der familie Andrenidae. Eigenverlag. Velden. (in German)

Smit, J., 2018. Identification key to the European species of the bee genus *Nomada* Scopoli, 1770 (Hymenoptera: Apidae), including 23 new species. Entomofauna. Vol.
