# Supplementary material for: Where and What Kind—A Better Understanding of Local and Landscape Features in Planning the Urban Flower Meadows for Supporting Bee Communities
Source: Ecol Evol. 2025 Jun 17;15(6):e71376. doi: 10.1002/ece3.71376 (PMC12171940; doi:10.1002/ece3.71376)

**
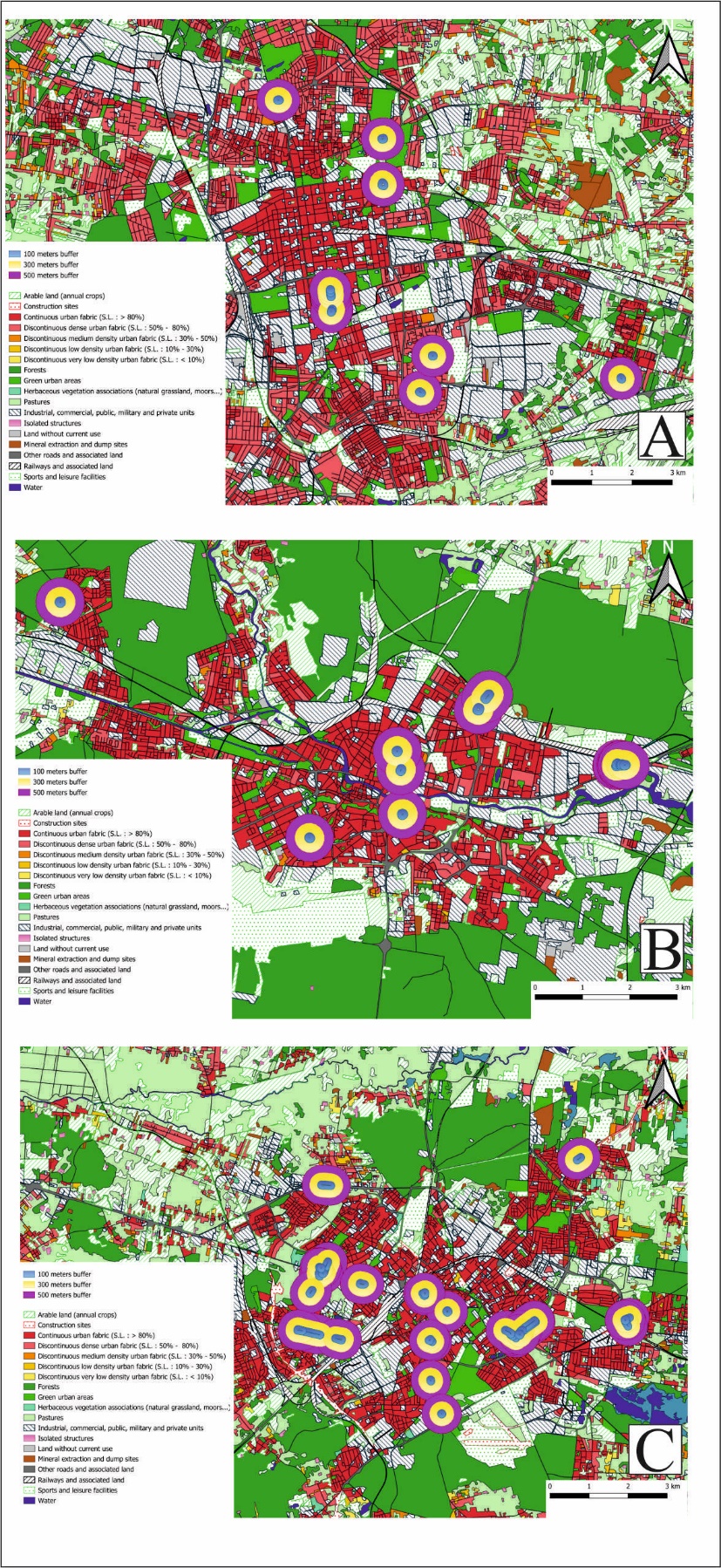
Figure S1**. Maps with 55 locations of sampled urban flower meadows with three buffer zones (100, 300, and 500 m) along with the land cover given according to the Urban Atlas landcover categories (given in Table S2). A - Łódź, B – Bydgoszcz, C - Białystok.

**Figure S2**. PCA ordination diagram for the six main PC axes for the 55 urban flower meadows' characterized by variable represented coverage (%) of different landscape classes (given in Table S2) in UFM surroundings. Analyses are given for 500-, 300-, and 100-m buffer zones.


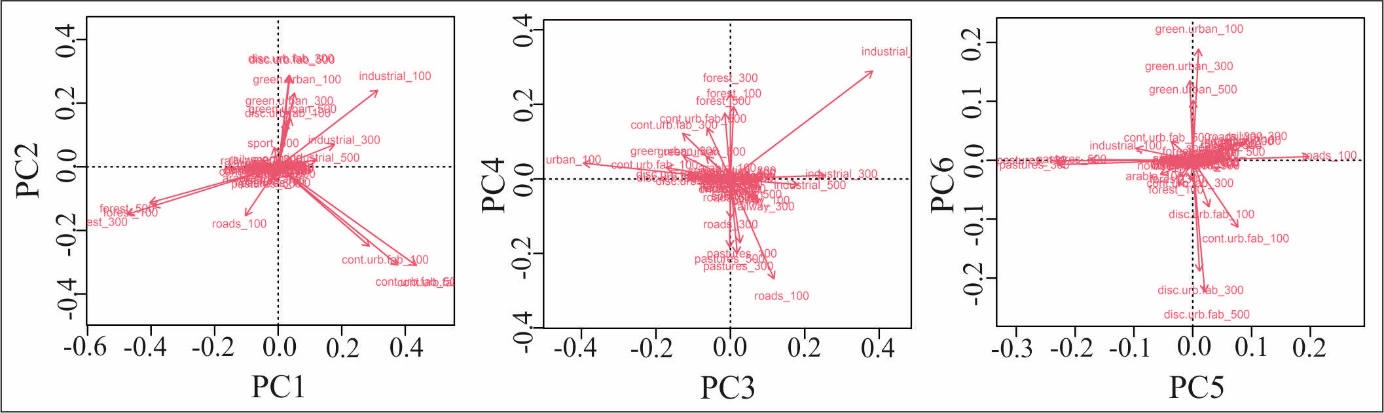


**Figure S3.** The PCA ordination diagram for seven flower color classes (white, yellow, white-yellow, pink, blue, purple, and red) was determined for each of the 55 UFMs.


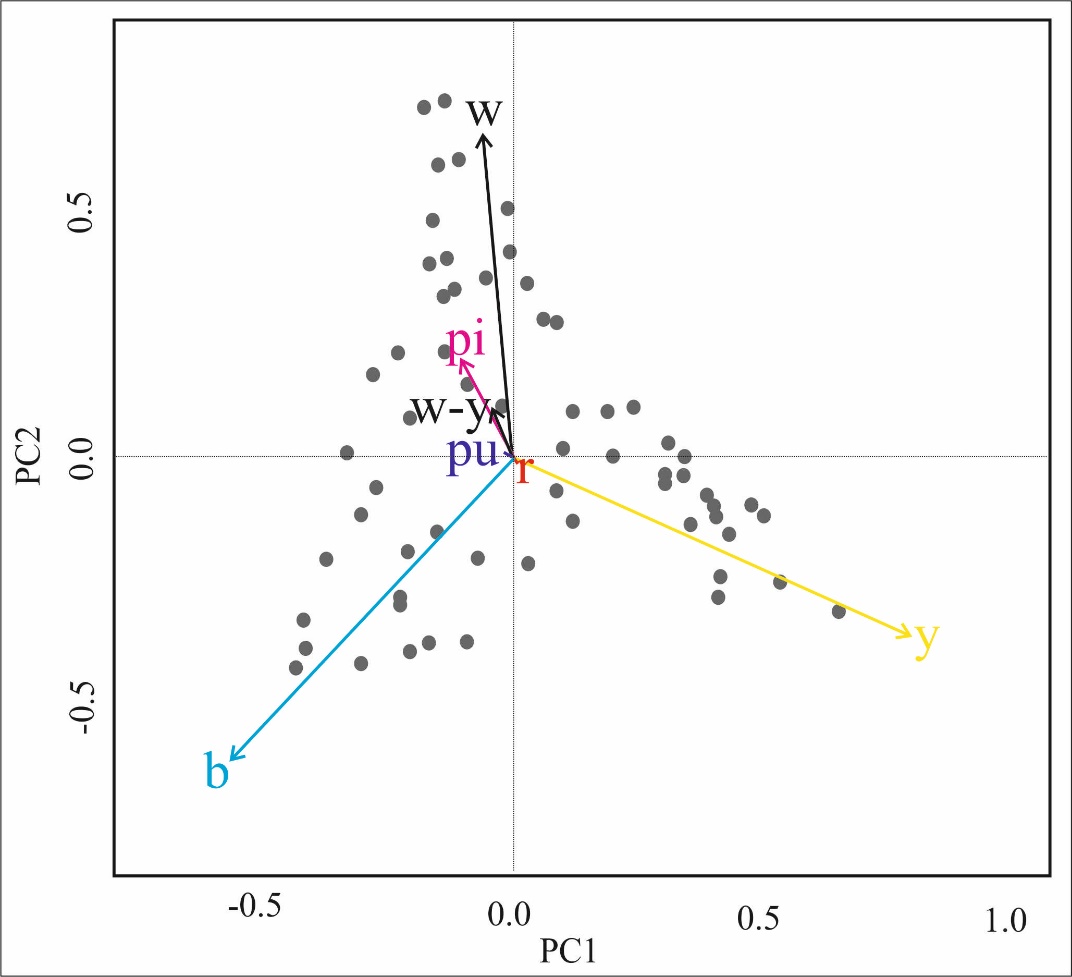


**Figure S4.** Histograms for each of the 22 local UFM variables concerning the flower resources.


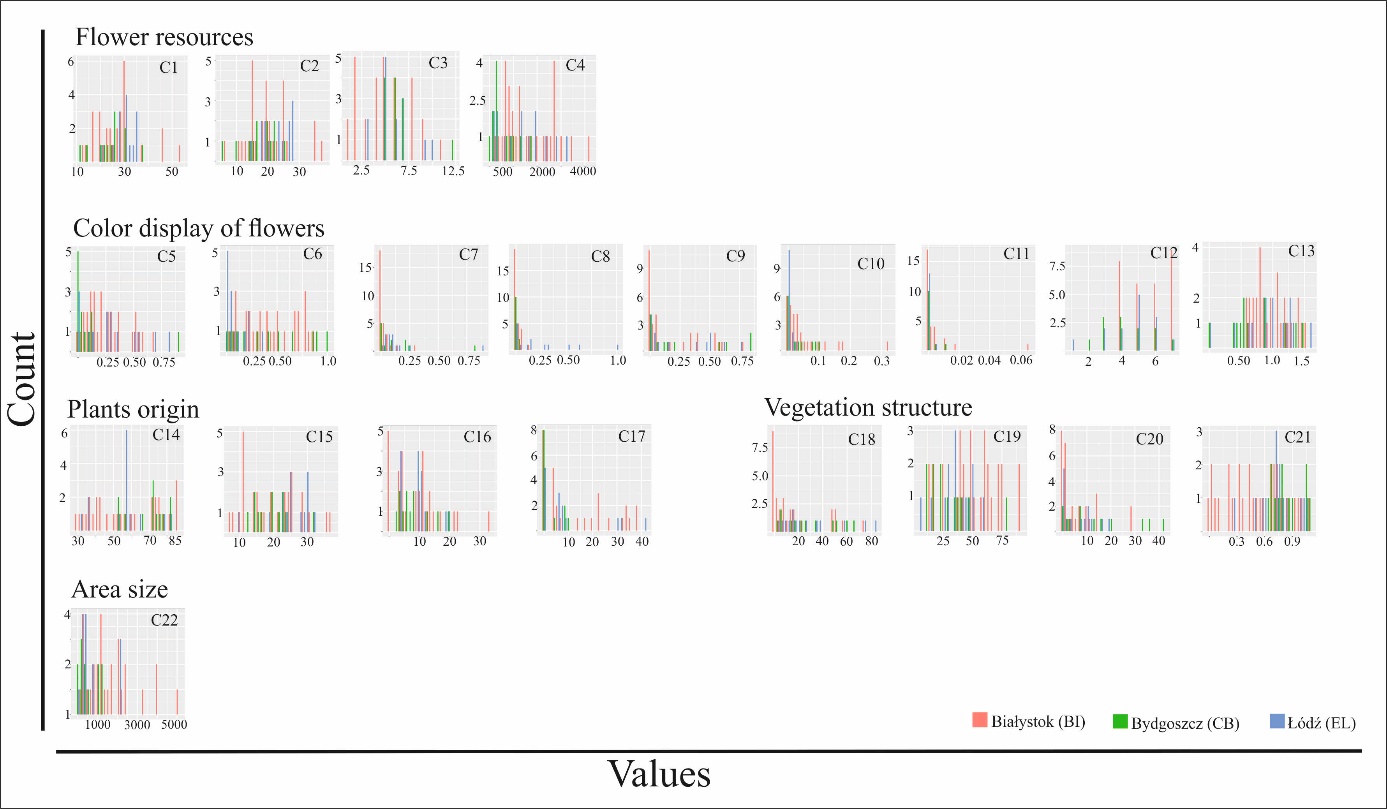


**Figure S5**. Bee species richness A) and abundance B) for each of the 55 UFMs located in three cities: BI – Białystok, CB – Bydgoszcz, EL – Łódź.


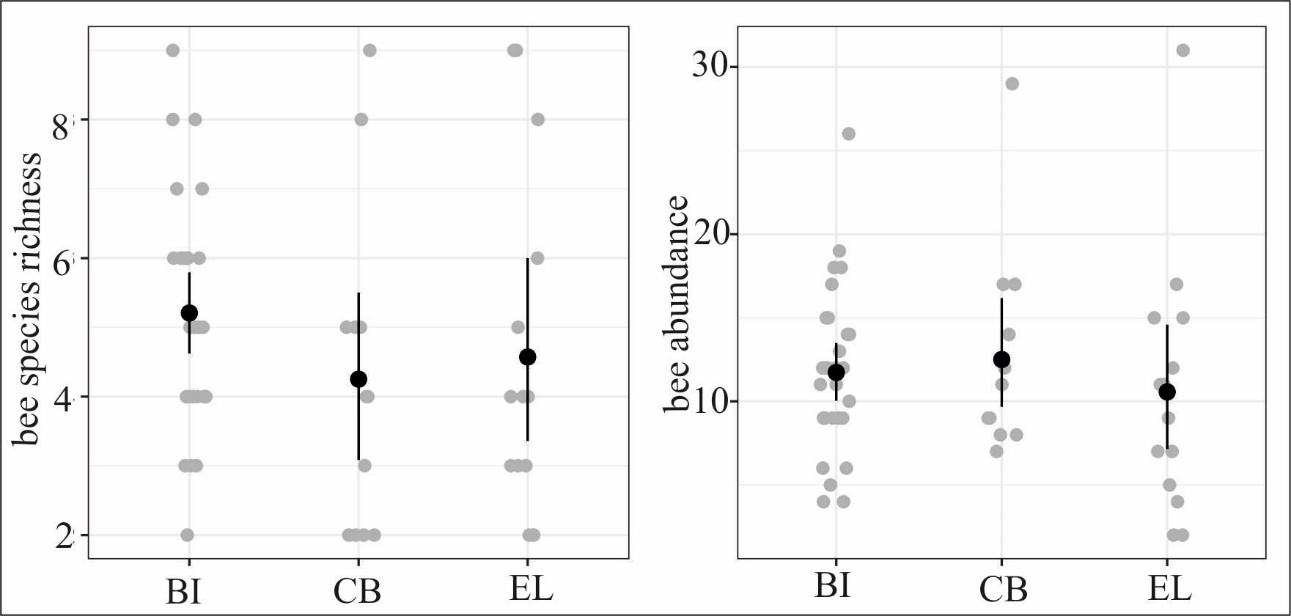


**Figure S6**. Ordination biplot diagram of redundancy analysis (RDA) for 55 UFMs in three cities (BI – Białystok, CB – Bydgoszcz, EL – Łódź). Vector labels refer to characteristics of the surroundings (Table S2 for explanation) for 500-, 300-, and 100-m buffer zones and for local UFM characteristics.


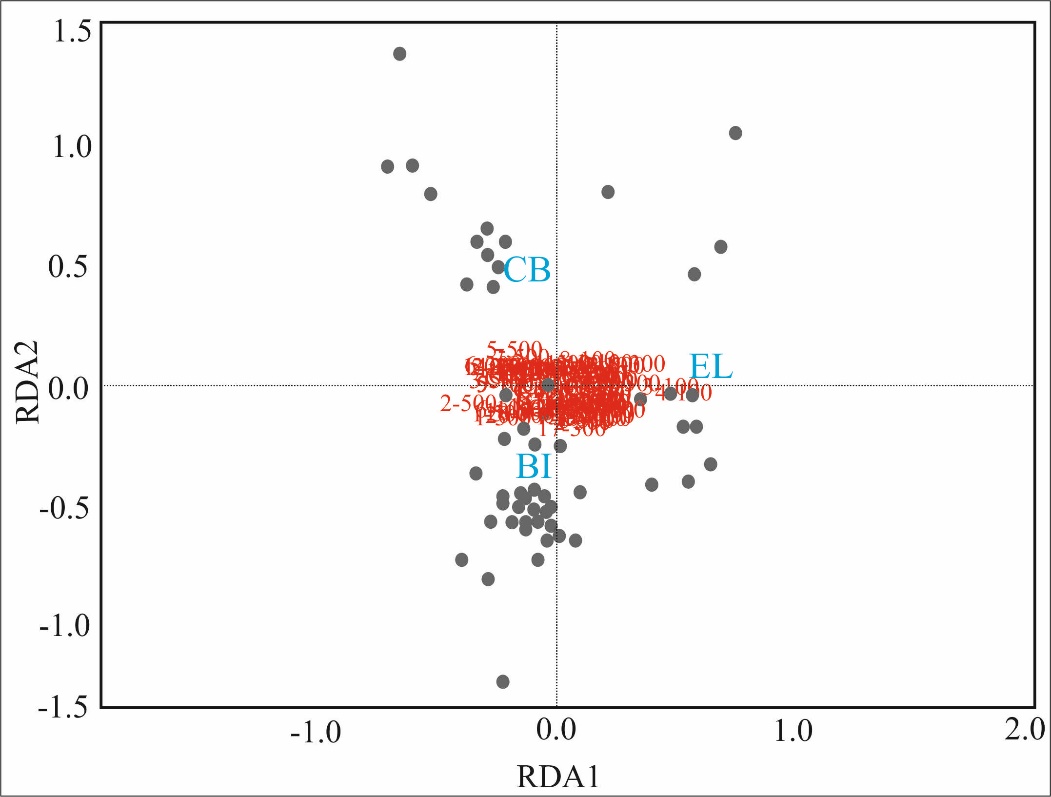

Supplement: Supplementary file 2 — Figures S1–S6. [file ECE3-15-e71376-s002.docx]
