# Supplementary material for: Where and What Kind—A Better Understanding of Local and Landscape Features in Planning the Urban Flower Meadows for Supporting Bee Communities
Source: Ecol Evol. 2025 Jun 17;15(6):e71376. doi: 10.1002/ece3.71376 (PMC12171940; doi:10.1002/ece3.71376)
Supplement: Supplementary file 3 — Tables S1–S4. [file ECE3-15-e71376-s001.docx]

**Supplements - Tables**

**Table S1.** Bee functional traits and their definitions are considered in this study (for Sobieraj-Betlińska & Twerd, 2024).

| Bee functional trait | Type | Definition |
| --- | --- | --- |
| Social behavior | Solitary | Each female solitary bee builds her own nest and provides food (pollen and nectar) for her offspring. |
|  | Eusocial | Females nest and breed into colonies. This group includes eusocial bumble bees *Bombus* and the primitively eusocial Halictidae. |
|  | Cleptoparasitic | Females do not build nests and lay their own eggs in cells provisioned by the bee host species. |
| Nest substrate | Soil | Females construct their nests beneath the surface of the soil. |
|  | Hive | Bee species that nest in hives in a variety of places, e.g. in hollow trees and stumps, or abandoned underground rodent burrows. This group includes non-parasitic bumblebees *Bombus* and several species of *Megachile*. |
|  | Cavity | This group comprises bee species whose females excavate their own nests in wood, as well as those that use pre-existing cavities such as in stones, wood, stems, or snail shells. |
| Floral specificity | Polylectic | Females visit a very wide spectrum of unrelated plant species for pollen. |
|  | Oligolectic | Females visit one plant family or genus for pollen. |
| Body length | Small | Bees with a body length less than 8 mm. |
|  | Medium | Bees with a body length of 8 to 15 mm. |
|  | Large | Bees with a body length greater than 15 mm. |

Sobieraj-Betlińska A., Twerd L. 2024. Are parks as favourable habitats for wild bees as wastelands in watercourse valleys of a large city? Urban Forestry & Urban Greening, 99: 128450. https://doi.org/10.1016/j.ufug.2024.128450

**Table S2.** The Urban Atlas landcover categories were used in this study. s.l. – surface land

| No. | Area type |
| --- | --- |
| 1 | Other urban wildflower meadow |
| 2 | Other roads and associated land |
| 3 | Pastures |
| 4 | Green urban areas |
| 5 | Forests |
| 6 | Continuous urban fabric (S.L. : >80%) |
| 7 | Discontinuous dense urban fabric (S.L.: 50%-80%) |
| 8 | Discontinuous medium density urban fabric (S.L.: 30%-50%) |
| 9 | Discontinuous low density urban fabric (S.L.: 10%-30%) |
| 10 | Discontinuous very low density urban fabric (S.L.: <10%) |
| 11 | Land without current use |
| 12 | Industrial, commercial, public, military and private units |
| 13 | Arable land (annual crops) |
| 14 | Sports and leisure facilities |
| 15 | Water |
| 16 | Mineral extraction and dump sites |
| 17 | Railways and associated land |
| 18 | Construction sites |
| 19 | Isolated structures |
| 20 | Herbaceous vegetation associations (natural grassland, moors...) |

**Table S3.** Checklist and functional traits of bee species recorded on UFMs in three Polish cities.

| **Family** |  | **Genus** | **Species** | **Author of species** | **Number of specimens** |  | **Social behavior** | **Nest substrate** | **Floral specificity** | **Body size length** |
| --- | --- | --- | --- | --- | --- | --- | --- | --- | --- | --- |
| Colletidae |  | *Colletes* | *daviesanus* | Smith, 1846 | 19 |  | solit | soil | poly | medium |
| Colletidae |  | *Colletes* | *fodiens* | (Fourcroy, 1785) | 10 |  | solit | soil | poly | medium |
| Colletidae |  | *Colletes* | *marginatus* | Smith, 1846 | 1 |  | solit | soil | poly | medium |
| Colletidae |  | *Colletes* | *similis* | Schenck, 1853 | 62 |  | solit | soil | poly | medium |
| Colletidae |  | *Hylaeus* | *communis* | Nylander, 1852 | 1 |  | solit | cav | poly | small |
|  |  |  |  |  |  |  |  |  |  |  |
| Andrenidae |  | *Andrena* | *dorsata* | (Kirby, 1802) | 4 |  | solit | soil | poly | medium |
| Andrenidae |  | *Andrena* | *flavipes* | Panzer, 1799 | 14 |  | solit | soil | poly | medium |
| Andrenidae |  | *Andrena* | *haemorrhoa* | (Fabricius, 1781) | 1 |  | solit | soil | poly | medium |
| Andrenidae |  | *Andrena* | *minutula* | (Kirby, 1802) | 1 |  | solit | soil | poly | small |
| Andrenidae |  | *Andrena* | *wilkella* | (Kirby, 1802) | 1 |  | solit | soil | oli | medium |
|  |  |  |  |  |  |  |  |  |  |  |
| Halictidae |  | *Halictus* | *rubicundus* | (Christ, 1791) | 0 |  | euso | soil | poly | medium |
| Halictidae |  | *Halictus* | *subauratus* | (Rossi, 1792) | 7 |  | euso | soil | poly | small |
| Halictidae |  | *Lasioglossum* | *calceatum* | (Scopoli, 1763) | 1 |  | euso | soil | poly | medium |
| Halictidae |  | *Lasioglossum* | *morio* | (Fabricius, 1793) | 1 |  | euso | soil | poly | small |
| Halictidae |  | *Lasioglossum* | *laticeps* | (Schenck, 1868) | 2 |  | euso | soil | poly | small |
| Halictidae |  | *Lasioglossum* | *nitidulum* | (Fabricius, 1804) | 1 |  | solit | soil | poly | small |
| Halictidae |  | *Lasioglossum* | *pauxillum* | (Schenck, 1853) | 4 |  | euso | soil | poly | small |
|  |  |  |  |  | 0 |  |  |  |  |  |
| Melittidae |  | *Dasypoda* | *hirtipes* | (Fabricius, 1793) | 6 |  | solit | soil | oli | medium |
| Melittidae |  | *Melitta* | *leporina* | (Panzer, 1799) | 17 |  | solit | soil | oli | medium |
|  |  |  |  |  |  |  |  |  |  |  |

**Table S3. cd**

| Family |  | Genus | Species | Author of  species | Number of specimens |  | Social behavior | Nest substrate | Floral specificity | Body size length |
| --- | --- | --- | --- | --- | --- | --- | --- | --- | --- | --- |
| Megachilidae |  | *Anthidiellum* | *strigatum* | Panzer, 1805 | 6 |  | solit | cav | oli | small |
| Megachilidae |  | *Anthidium* | *manicatum* | Linnaeus, 1758 | 33 |  | solit | cav | poly | medium |
| Megachilidae |  | *Anthidium* | *oblongatum* | Illiger, 1806 | 3 |  | solit | cav | poly | medium |
| Megachilidae |  | *Chelostoma* | *campanularum* | Kirby, 1802 | 1 |  | solit | cav | oli | small |
| Megachilidae |  | *Chelostoma* | *rapunculi* | Lepeletier, 1841 | 3 |  | solit | cav | oli | medium |
|  |  |  |  |  |  |  |  |  |  |  |
| Megachilidae |  | *Heriades* | *truncorum* | Linnaeus, 1758 | 2 |  | solit | cav | oli | small |
|  |  |  |  |  |  |  |  |  |  |  |
| Megachilidae |  | *Hoplitis* | *adunca* | Panzer, 1798 | 9 |  | solit | cav | oli | medium |
| Megachilidae |  | *Hoplitis* | *leucomelana* | Kirby, 1802 | 1 |  | solit | cav | poly | small |
|  |  |  |  |  |  |  |  |  |  |  |
| Megachilidae |  | *Megachile* | *circumcincta* | Kirby, 1802 | 1 |  | solit | soil | poly | medium |
| Megachilidae |  | *Megachile* | *ericetorum* | Lepeletier, 1841 | 2 |  | solit | hiv | oli | medium |
| Megachilidae |  | *Megachile* | *lagopoda* | (Linnaeus, 1761) | 14 |  | solit | soil | poly | large |
| Megachilidae |  | *Megachile* | *rotundata* | Fabricius, 1793 | 5 |  | solit | hiv | poly | medium |
| Megachilidae |  | *Megachile* | *willughbiella* | Kirby, 1802 | 13 |  | solit | cav | poly | medium |
|  |  |  |  |  |  |  |  |  |  |  |
| Megachilidae |  | *Osmia* | *aurulenta* | Panzer, 1799 | 1 |  | solit | cav | poly | medium |
| Megachilidae |  | *Osmia* | *brevicornis* | Fabricius, 1798 | 1 |  | solit | cav | oli | medium |
| Megachilidae |  | *Osmia* | *niveata* | Fabricius, 1804 | 1 |  | solit | cav | oli | medium |
|  |  |  |  |  |  |  |  |  |  |  |
| Apidae |  | *Anthophora* | *bimaculata* | Panzer, 1798 | 1 |  | solit | soil | poly | medium |
| Apidae |  | *Anthophora* | *furcata* | Panzer, 1798 | 1 |  | solit | soil | oli | medium |
| Apidae |  | *Anthophora* | *plumipes* | (Pallas, 1772) | 1 |  | solit | soil | poly | medium |
|  |  |  |  |  |  |  |  |  |  |  |
| Apidae |  | *Apis* | *mellifera* | Linnaeus, 1758 | 258 |  | euso | hiv | poly | medium |

| Family |  | Genus | Species | Author of  species | Number of specimens |  | Social behavior | Nest substrate | Floral specificity | Body size length |
| --- | --- | --- | --- | --- | --- | --- | --- | --- | --- | --- |
| Apidae |  | *Bombus* | *bohemicus* | Seidl, 1838 | 1 |  | clep | clep | clep | medium |
| Apidae |  | *Bombus* | *hypnorum* | Linnaeus, 1758 | 3 |  | euso | hiv | poly | large |
| Apidae |  | *Bombus* | *hortorum* | Linnaeus, 1758 | 6 |  | euso | hiv | poly | large |
| Apidae |  | *Bombus* | *humilis* | Illiger, 1806 | 2 |  | euso | hiv | poly | large |
| Apidae |  | *Bombus* | *jonellus* | Kirby, 1802 | 1 |  | euso | hiv | poly | medium |
| Apidae |  | *Bombus* | *lapidarius* | Linnaeus, 1758 | 57 |  | euso | hiv | poly | large |
| Apidae |  | *Bombus* | *pascuorum* | Scopoli, 1763 | 27 |  | euso | hiv | poly | medium |
| Apidae |  | *Bombus* | *pratorum* | Linnaeus, 1758 | 5 |  | euso | hiv | poly | medium |
| Apidae |  | *Bombus* | *ruderarius* | Müller, 1776 | 6 |  | euso | hiv | poly | medium |
| Apidae |  | *Bombus* | *sylvarum* | Linnaeus, 1758 | 3 |  | euso | hiv | poly | medium |
| Apidae |  | *Bombus* | *Terrestribombus* complex | Vogt, 1911 | 32 |  | euso | hiv | poly | large |
|  |  |  |  |  |  |  |  |  |  |  |

**Table S3. cd**

Functional traits: social behavior (solit = solitary, euso = eusocial, clep = cleptoparasitic); nest substrate (cav = cavity, soil = soil, hiv = hive); floral specificity (oli = oligolectic, poly = polylectic); body size (small, medium, large).

**Table S4.** Chao estimators of three diversity estimates: number of species (species richness) and Shannon’s and Simpson’s indices with standard errors and confidence limits.

| Assemblage | Diversity |  | Observed | Estimator | s.e. | LCL | UCL |
| --- | --- | --- | --- | --- | --- | --- | --- |
| Bialystok | Species richness |  | 35.000000 | 50.084987 | 19.6801765 | 35.000000 | 88.657424 |
| Bialystok | Shannon diversity |  | 13.569474 | 14.547438 | 0.9572499 | 12.671263 | 16.423613 |
| Bialystok | Simpson diversity |  | 7.343201 | 7.468868 | 0.6231451 | 6.247526 | 8.690210 |
|  |  |  |  |  |  |  |  |
| Bydgoszcz | Species richness |  | 21.000000 | 41.128012 | 21.3132700 | 21.000000 | 82.901254 |
| Bydgoszcz | Shannon diversity |  | 7.154418 | 8.060325 | 1.1078347 | 5.889009 | 10.231641 |
| Bydgoszcz | Simpson diversity |  | 4.098156 | 4.176578 | 0.4610854 | 3.272867 | 5.080289 |
|  |  |  |  |  |  |  |  |
| Lodz | Species richness |  | 25.000000 | 37.020395 | 10.9299839 | 25.000000 | 58.442769 |
| Lodz | Shannon diversity |  | 7.503899 | 8.568043 | 1.2302444 | 6.156808 | 10.979278 |
| Lodz | Simpson diversity |  | 3.480566 | 3.538699 | 0.5001326 | 2.558457 | 4.518941 |
